# Case Report: Two Cases of Pediatric Thrombotic Thrombocytopenic Purpura Treated With Combined Therapy

**DOI:** 10.3389/fped.2021.743206

**Published:** 2021-11-02

**Authors:** Costanza Tripiciano, Paola Zangari, Mauro Montanari, Giovanna Leone, Laura Massella, Lucia Garaboldi, Michela Massoud, Stefano Lancellotti, Luisa Strocchio, Emma Concetta Manno, Paolo Palma, Tiziana Corsetti, Matteo Luciani

**Affiliations:** ^1^Unit of Clinical Immunology and Vaccinology, Academic Department of Pediatrics, Bambino Gesù Children's Hospital, IRCCS, Rome, Italy; ^2^Academic Department of Pediatrics, University of Rome Tor Vergata, Rome, Italy; ^3^Unit of Transfusion Medicine, Department of Diagnostic and Laboratory Medicine, Bambino Gesù Children's Hospital, IRCCS, Rome, Italy; ^4^Department of Pediatric Subspecialties, Division of Nephrology, Bambino Gesù Children's Hospital, IRCCS, Rome, Italy; ^5^Unit of Hospital Pharmacy, Bambino Gesù Children's Hospital, IRCCS, Rome, Italy; ^6^Department of Pediatric Hemato-Oncology and Cell and Gene Therapy, Bambino Gesù Children's Hospital, IRCCS, Rome, Italy; ^7^Haemorrhagic and Thrombotic Diseases Service, Area of Hematology, Fondazione Policlinico Universitario “A. Gemelli”, IRCCS, Rome, Italy; ^8^Chair of Pediatrics, Department of Systems Medicine, University of Rome “Tor Vergata”, Rome, Italy

**Keywords:** pediatrics, TTP (thrombotic thrombocytopenic purpura), plasma exchange (PEX), immunosuppressive therapy, caplacizumab

## Abstract

Thrombotic thrombocytopenic purpura (TTP) is a thrombotic microangiopathy caused by a severely reduced activity of the von Willebrand factor-cleaving protease ADAMTS13. Over 95% of TTPs are acquired, due to autoantibody inhibitors. In children, acquired TTP is a very rare, life-threatening disease. To date, no consensus exists on the treatment strategy of pediatric TTP. We report the cases of two pediatric patients with a diagnosis of TTP, successfully treated with a combination of various therapeutic approaches. Although the patients complained of different sets of symptoms, laboratory data showed Coombs negative hemolytic anemia, renal impairment, and low platelet count in both cases. The diagnosis of acquired TTP was supported by the PLASMIC score and confirmed by the reduction of the ADAMTS13 activity and the presence of anti-ADAMTS13 antibodies. Intravenous immunoglobulin, corticosteroids, and plasma exchange (PEX) were performed without delay. As soon as available, caplacizumab was added to the therapy, with a prompt normalization of platelet count. Nevertheless, ADAMTS13 activity was persistently low, and anti-ADAMTS13 antibodies level was high; thus, a course of rituximab was administered, with persistent normalization of laboratory findings. No adverse events were observed during the treatment. In our experience, the combined use of PEX, caplacizumab, and immunosuppressive therapy during the acute phase of the disease is safe and may have a significant impact on the prognosis with successful clinical outcome and decrease in life-threatening events.

## Introduction

Thrombotic thrombocytopenic purpura (TTP) is a thrombotic microangiopathy caused by reduced activity of the von Willebrand factor (VWF)-cleaving protease ADAMTS13. TTP can be acquired, due to autoantibody inhibitors, or hereditary, due to homozygous or compound heterozygous ADAMTS13 mutations (1). In adults, the incidence of acquired TTPs is ~3 cases per 1 million per year, and it accounts for >95% of all ADAMTS-13-deficient TTP cases. However, the prevalence of TTP could vary worldwide, as demonstrated by Von Krogh et al. (2), who discovered a much higher prevalence of congenital TTP in a health district in Norway. In contrast to adults, in newborn infants and young children, hereditary TTP is more common than acquired TTP, which is very rare in children, with an incidence of ~1 per 10 million per year in children <18 years old (~30-fold less common than in adults) (3). TTP can be life threatening, and ~34% of patients who survived an acute episode present a relapsing disease (4). Several therapies are available for treatment of TTP, including plasma exchange (PEX) and immunosuppressive agents (5–7). Recently, caplacizumab, a humanized nanobody targeting von Willebrand factor (VWF), has been used in the treatment of TTP (8–10), and randomized clinical trials have confirmed its efficacy in the adult population (11). Despite increasing knowledge on the pathogenesis of TTP in the last years, the therapeutic approach varies significantly, due to the lack of high-quality evidence to support strong recommendations (12). Moreover, no guidelines or clinical trials for the pediatric population are available at present.

Herein, we report the cases of two pediatric patients treated with PEX, immunosuppressive therapy, and caplacizumab in the acute phase (Table 1).

**Table 1:** Demographic and outcome factors in our two cases.

	**Patient 1**	**Patient 2**
**Demographic**	
Age (years)	16	15
Sex	F	F
Ethnicity	Caucasian	Caucasian
ADAMTS13 activity on admission (%)	<1%	<1%
Anti-ADAMTS13 antibodies on admission (U/ml)	66.9	130
Number of PEX therapies	15	12
Time from PEX start to the first caplacizumab infusion (days)	4	1
Time from caplacizumab start to the first rituximab infusion (days)	23	28
**Outcomes**	
Time to platelet count normalization (days)	10	6
Time to ADAMTS13 activity >10% (days)	42	56
Time to PLT normalization after first PEX (days)	6	5
Time to PLT normalization after I dose of caplacizumab (days)	3	5
Time to ADAMTS13 >10% after first PEX (days)	39	35
Time to ADAMTS13 >10% after first rituximab (days)	14	6
Duration of caplacizumab therapy (days)	42	39
Complications	No	No
Death	No	No
Adverse effects to therapy	No	No

## Case 1

**Figure 1:**
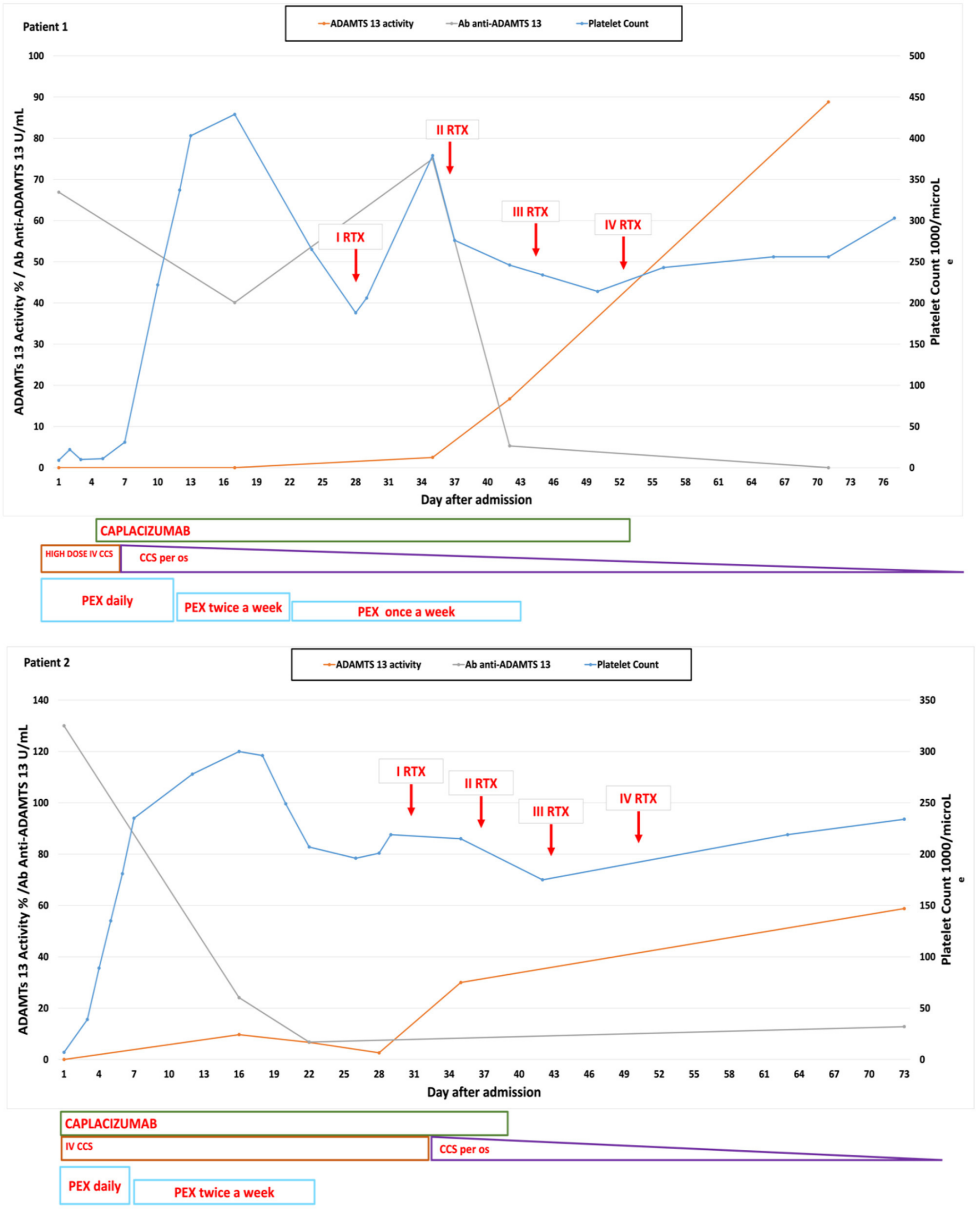
Graphs showing the platelet count, ADAMTS13 activity, and the anti-ADAMTS13 antibody trend correlated with the administered therapies in our two cases.

A 16-year-old overweight girl was admitted to the emergency department due to a chin trauma having occurred after a syncope. The girl complained of nausea, headache, and vomiting in the past days. Her past medical history and family history was unremarkable. On admission, she had fever and laboratory data showed mild renal impairment (serum creatinine 1.16 mg/dl), hemolytic anemia (lactate dehydrogenase 2,238 U/L, hemoglobin 5.4 g/dl, indirect bilirubin 1.54 mg/dl, reticulocyte count 31.6 × 10^4^/μl, and negative Coombs test), low platelet count (7 × 10^9^/L), and schistocytes (>1%) in the peripheral blood smear. Autoimmune cytopenia and hematological malignancies were ruled out. The screening for infectious disease showed a past EBV infection and an acute urinary tract infection caused by *Escherichia coli*. Reactive C protein (RCP) was 1.04 mg/dl (normal value <0.5), and erythrocyte sedimentation rate (ESR) was 129 mm (normal value 0–15). Hemolytic uremic syndrome (HUS) was unlikely due to the not suggestive history with negative stool cultures and normal C3 complement fraction. During hospitalization, she presented with a transient episode of dysarthria and confusion with negative cerebral computed tomography scan. No signs of cardiac involvement were detected (normal ECG and echocardiogram). Clinical and laboratory data suggested the diagnosis of TTP, supported by the evaluation of the PLASMIC score, which was 7 (high risk) (13) and later confirmed by the reduction in the ADAMTS13 activity (<1%). Congenital TTP was unlikely because of the age of the patient and the unremarkable past medical history, and the diagnosis of acquired TTP was confirmed by the presence of anti-ADAMTS13 antibodies (66.9 U/ml, normal value <15 U/ml). In addition, we performed immunological tests, which showed an increase in the atypical B memory cells, according to the autoimmune process. Intravenous high-dose immunoglobulins and corticosteroids were immediately performed. Daily PEX was started as soon as available, associated with corticosteroids and low molecular weight heparin when platelet count was >50 × 10^9^/L. Caplacizumab (10 mg/day) was added 4 days later, and it was well-tolerated. Three days after starting caplacizumab, the platelet count gradually increased. During the following weeks, anti-ADAMTS13 antibodies remained persistently high, and ADAMTS13 activity was persistently low; thus, a four-dose course of rituximab (375 mg/m^2^/week) was started. At the end of the course, ADAMTS13 activity increased up to 64%, and anti-ADAMTS13 antibodies levels concurrently decreased to 5.3 U/ml (Figure 1). To the date, 77 weeks after withdrawal of the therapy, patient is in good clinical condition, and her platelet count is within the normal range.

## Case 2

A 15-year-old obese girl was admitted to our hospital for vomiting and petechial eruptions of the extremities. Her medical history was characterized by obesity and vitamin D deficiency. Her father was affected by severe hypothyroidism. Blood exams showed hemolytic anemia (Hb 7.3 g/dl, indirect bilirubin 7.10 mg/dl, lactate dehydrogenase 1,953 U/L, reticulocyte count 56.3 × 10^4^/μl, negative Coombs test), schistocytes in the peripheral blood smear, a severe thrombocytopenia (7 × 10^9^/L), and a mild renal impairment (creatinine 1.07 mg/dl). Hematological disorders, autoimmune cytopenia, and HUS were excluded. A screening for infectious disease was also performed, which showed a past EBV and CMV infection. No clinical signs of neurological involvement were shown. Echocardiograph and cardiac enzymes were normal. RCP was 3.72 mg/dl (normal value <0.5), and ESR was 102 mm (normal value 0–15). The immunological tests showed an expansion of the CD19 cells. We considered the diagnosis of TTP; thus, we performed the PLASMIC score, which was 6 (high risk). The ADAMTS13 activity was <1%, and the level of the anti-ADAMTS13 antibodies was 130 U/ml. The patient was treated with intravenous high-dose immunoglobulin infusions, daily PEX, oral prednisolone (1 mg/kg/day), and low molecular weight heparin. In contrast to the first case, caplacizumab (10 mg/day) was immediately administered. A significant increase in platelet count was observed after two administrations, and in 5 days, her platelet count was normal. No adverse events were observed during the treatment with caplacizumab. After 1 month of therapy, ADAMTS13 activity was persistently low, and anti-ADAMTS13 antibody level was high. For this reason, as in the previous case, a course of rituximab (375 mg/m^2^/week) was administered. Once the rituximab course was completed, ADAMTS13 activity was 58% and anti-ADAMTS13 antibodies were undetectable. Platelet count remained normal even when PEX and caplacizumab were discontinued (Figure 1). At present, 38 weeks after completing the rituximab course, the patient is in good health condition with normal platelet count.

In both cases, organ damage markers were monitored daily during the acute phase, whereas ADAMTS13 activity and anti-ADAMTS13 antibodies were measured every 7–15 days. ADAMTS13 activity was quantified by fluorescence resonance energy transfer (FRET) assay, while the anti-ADAMTS13 antibodies were detected with a simplified enzyme-linked immunosorbent assay (ELISA). PEX was discontinued after platelet count reached a value >150 × 10^9^ for 2 days. Caplacizumab was administered intravenously the first time and, afterward, subcutaneously. When clinical stability and improvement of blood test results were reached, patients were discharged, and caplacizumab was administered at home with a weekly clinical and laboratory follow up. Caplacizumab has been withdrawn after ADAMTS13 activity recovered a value >10%, as suggested by many experts (14).

## Discussion

TTP is included in the group of microangiopathic hemolytic anemias and occurs predominantly in the adult population (3, 15). In children, TTP is extremely rare, and at the onset, differential diagnosis from others thrombotic microangiopathies may be very challenging (16). The most important differential diagnosis is congenital TTP also known as Upshaw–Schulman syndrome. A rapid identification is critical to outcome, as treatment delay is potentially life threatening (17).

Here, we reported two cases of TTP in adolescents with a similar successful therapeutic approach. Current evidence suggests that conventional treatment with PEX can improve survival rates from 20 to 80% (6) by removing anti-ADAMTS13 antibodies, ADAMTS13 immune complexes, and replacing ADAMTS13 activity. Most patients with acquired TTP receive immunosuppressive therapy in addition to PEX. This practice is supported by the autoimmune nature of the disease. Retrospective and prospective studies employing rituximab along with continued daily PEX reported encouraging results (18, 19). Owattanapanich et al. (20) demonstrated that rituximab offered high efficacy for the prevention of relapses and was associated with a lower mortality rate in cases of acquired TTP, especially in patients receiving it in an acute phase. Rituximab is an anti-CD20-chimeric monoclonal antibody, and its efficacy is due to B-cell depletion, which allows suppression of anti-ADAMTS13 autoantibody production. In our cases, immunological characterization is consistent with an autoimmune process, and this fact strongly supported the use of an anti-CD20 therapy. Caplacizumab is a humanized bivalent nanobody that consists of two identical, genetically linked, humanized building blocks targeting the A1 domain of the von Willebrand factor and inhibiting the interaction between the von Willebrand factor and platelets. It was initially used as a second-line treatment (8–10, 21). However, caplacizumab was recently approved for the treatment of TTP in the adult setting as first-line therapy for TTP (11). In April 2020, caplacizumab was also approved for the treatment of adolescents of 12 years of age and older weighing at least 40 kg experiencing an episode of acquired thrombotic thrombocytopenic purpura. Despite Cablivi has not been already approved for children at the time of the first patient, in this case, caplacizumab was administrated off label because she was not much younger than 18 years and had an elevated BMI; therefore, she was comparable with an adult. Although, Goshua et al. (22) showed that caplacizumab does not appear to be cost effective as the current drug pricing, it represents an important advance in TTP therapy. Indeed, recent studies showed that the use of caplacizumab during the acute phase is able to prevent unfavorable outcomes, decreasing thromboembolic event rate, and the number of days of PEX and hospitalization (23–25). Even though PEX is still considered crucial in the treatment of TTP, successful outcomes with the use of multiple treatment strategies without PEX were recently reported, suggesting the possibility that PEX may be reserved to patients with very severe or refractory disease (26–28), even though much more evidence from specific clinical trials is needed to support this hypothesis.

Based on our experience, we could suggest that caplacizumab is well-tolerated in pediatric patients affected by TTP. In both our cases, no hemorrhagic events were recorded as well as in the pediatric patients reported by Dutt et al. (24). Although caplacizumab does not modify the underlying immune pathophysiology of TTP, a prompt administration appears to impact the timely recovery of platelet count, as confirmed by the time to platelet count normalization in our cases (Table 1) and the risk of life-threatening events.

In conclusion, we speculate that the combined use of caplacizumab and immunosuppressive therapy in the acute phase may have a significative impact on the prognosis. The combination of various therapeutic approaches, such as blocking VWF-mediated platelet adhesion and aggregation in the microcirculation, inhibiting the inflammatory pathways, and suppressing the production of anti-ADAMTS13 autoantibodies, is crucial to obtain a significant response to treatment and a lasting maintenance of clinical and laboratory remission. Although our experience on a very limited number of patients does not allow drawing any firm conclusions regarding a potential advantage in using a caplacizumab-based, PEX-free approach, in our opinion, these data might suggest the efficacy and safety of this strategy in the pediatric population, which could support further investigations on this topic.

## Data Availability Statement

The original contributions presented in the study are included in the article/supplementary material, further inquiries can be directed to the corresponding author.

## Ethics Statement

All procedures performed in the study were in accordance with the ethical standards of the institutional research committee and with the 1964 Helsinki declaration. Written informed consent, following standard ethical procedures, with approval of the Children's Hospital Bambino Gesù Ethical Committee, was obtained from parents of the patients.

## Author Contributions

CT, PZ, and LM collected data and were involved in the conception of the paper. CT wrote the first draft of the manuscript. EM, LM, MMa, and LS wrote the sections of the manuscript. PP, SL, MMo, GL, LG, and TC made critical revisions on the manuscript. All authors have contributed to the manuscript, reviewed, and approved the submitted version.

## Conflict of Interest

The authors declare that the research was conducted in the absence of any commercial or financial relationships that could be construed as a potential conflict of interest.

## Publisher's Note

All claims expressed in this article are solely those of the authors and do not necessarily represent those of their affiliated organizations, or those of the publisher, the editors and the reviewers. Any product that may be evaluated in this article, or claim that may be made by its manufacturer, is not guaranteed or endorsed by the publisher.

## Abbreviations

ADAMTS13: A disintegrin and metalloproteinase with a thrombospondin type 1 motif
member 13; TTP: Thrombotic thrombocytopenic purpura
VWF: von Willebrand factor
PEX: plasma exchange
HUS: Hemolytic uremic syndrome
RCP: Reactive C protein
ESR: Erythrocyte sedimentation rate.
